# Dielectric Behavior of the Film Formed on Mica Cleaved in Moist Air

**DOI:** 10.6028/jres.068A.017

**Published:** 1964-04-01

**Authors:** S. Ruthberg, L. Frenkel

## Abstract

Water adsorbed on a freshly peeled mica crystal causes the loss tangent, *D*, to increase by 1 to 2 orders of magnitude. The nature of the film is investigated as a function of relative humidity by the measurement of *D* for the frequency range 100 to 50,000 c/s with a capacitor comprising concentric, parallel, circular electrodes of different diameter on opposite sides of the dielectric sheet. This geometry is then analyzed as consisting of the two regions of that within the plates where the electric field, *E*, is normal to the dielectric plane and that at the edge where tangential *E* exists. The first is considered in terms of an equivalent circuit for a two layered dielectric. The second is considered in terms of transmission line concepts. It is predicted and verified that the adsorbed film causes the first component to vary as 1/*t* and the second as √*t* where *t* is the thickness of the crystal. Numerical solutions are used to derive the behavior of *D, R*, and *C* of the adsorbed film itself. *D* for the normal direction is ~0.4 and follows a frequency dependency like fresh snow. Resistivities normal to and parallel to cleavage are considerably different while *C* is much less than expected for a surface film. It is suggested that the surface film is not continuous but instead is localized in patches.

## 1. Introduction

The nature of adsorbed water on cleaved mica has long been of interest for its effects on the insulation quality of the material and on the force necessary to split the crystal [1–4].^1^ Previous measurements of electrical losses in the presence of moisture have shown the effect to be transient and of a complex behavior [5–13].

The present paper reports results of a study of the a-c properties of the adsorbed film. The effort was to study the conductivity parallel to, as well as normal to, the cleavage plane and to derive a further understanding of the structure of the film itself.

Our examination of the material consisted of the measurement of the loss-tangent for the frequency range 100 to 50,000 c/s, for the relative humidity range of about 6 to 60 percent, and for specimens cleaved to a number of thicknesses. Various capacitor arrangements were used, with one in particular consisting of a sheet of the dielectric with one large circular electrode and one small circular electrode on opposite faces. The procedures used and typical results obtained are discussed in section 2. In section 3, the asymmetrical capacitor is analyzed in terms of two regions. One is that between the electrodes where the *E* field is always perpendicular to the cleavage plane and the other is that at the edge of the electrodes where there is a parallel component of *E.* The behavior of the domain within the normal field is analyzed as a two-layered dielectric, mica sheet plus surface film. The region at the edge is considered in terms of transmission line concepts. From these considerations, relationships are derived for dependency of loss tangent on thickness of the dielectric, for a separation of normal and parallel effects, and for deduction of adsorbed film characteristics. The applicability of these expressions was tested by studying the change in loss tangent with thickness and the amount of field fringing as is reported in section 4. Finally, some conclusions on the nature of the film itself are presented in section 5.

## 2. Experimental Procedures and Nature of Loss Tangent

### 2.1. Specimens

Specimens used for this work were obtained from the Defense Materials Service of the General Services Administration and were selected for quality (ASTM visual Classification V–1 to V–4 [14]) by the mica experts of that organization. Original samples varied in thickness from about 0.15 to 0.38 mm. All had been well aged since last cleaved.

### 2.2. Apparatus

**Figure 1:**
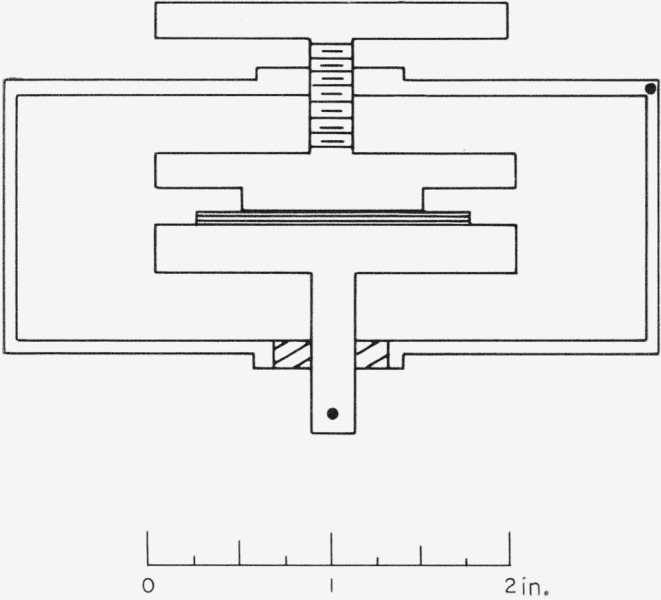
Capacitor geometry. Plates are to scale; mica thickness is not.

A micrometer-driven dielectric sample holder of the Hartshorn type (General Radio Co. Model 1690–A) was modified by placing a cap over the upper movable plate which reduced the 2 in. diam to 1 in. diam. The resultant capacitor was as sketched in figure 1. The upper plate was driven onto the mica sheet so that the specimen was clamped between the smaller upper and larger lower plates. Loss tangent and capacity were determined by substitution with a Schering bridge and associated circuitry (General Radio Type 1610–A Capacitance Measuring Assembly) for frequencies of 100 to 50,000 c/s. Capacitance readings with this bridge are accurate to about ±1 pf in substitution measurements, and loss tangent readings are accurate to about ±5×10^−5^ or to ±2 percent, whichever is larger.

The capacitor head was placed in a humidity chamber. Relative humidity was varied from about 6 to 60 percent as measured with a surface film hygrometer (Aminco). Mica sheets were cleaved while within the chamber and were then moved at constant humidity directly into the capacitor head.

### 2.3. Correction for Residual Airgap

**Figure 2:**
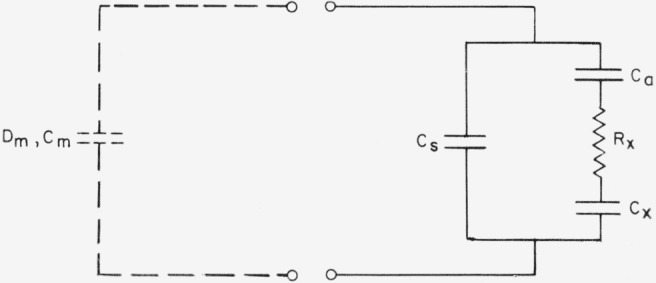
Equivalent circuit for capacitor assembly. The cleaved mica lamina is represented here by the series combination of *R_x_* and *C_x_.*

Because of the existence of residual airgaps between the sample holder and the specimen, it was necessary to correct the measured values of capacitance and *D*. The assumed equivalent circuit for the sample holder is shown in figure 2. *C_m_* and *D_m_* are the measured values of capacitance and loss tangent of the holder with specimen, *C_s_* represents the stray capacitance, and *C_a_* represents the residual airgap. The mica specimen is represented as the general series combination of *R_x_* and *C_x_.* With the assumption that the effective *D* for the mica with surface film is very much less than one (as shown later to be true),

$$\frac{1}{C_{x}}=\frac{1}{C_{m}-C_{s}}-\frac{1}{C_{a}}.$$ (1)

**Figure 3:**
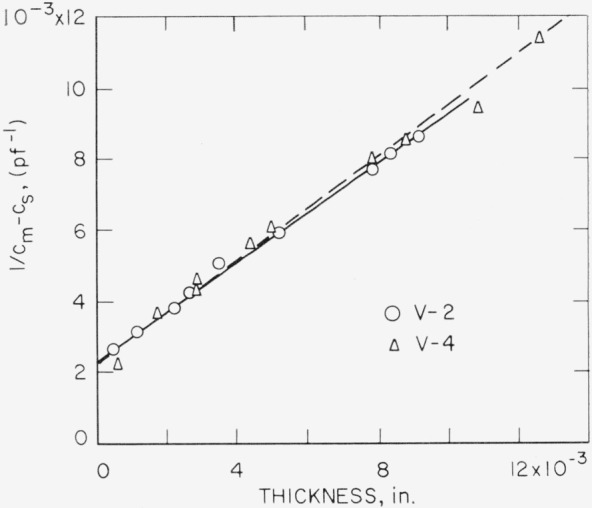
1/(*C*_m_—*C*_s_) versus thickness for cleaved specimens of V–2 India Ruby and V–4 Brazil Ruby. For Rh~40% and frequency of 1000 c/s.

But *C_x_* ∝ *ϵ*/*t*, where *t* is thickness and *ϵ* is the dielectric constant of the specimen. If micas of various thicknesses are inserted into the holder, *C_a_* and *ϵ* can be determined from eq (1) provided *C_a_* and *ϵ* are constant. With *C_s_* determined by substitution as 68 pf, figure 3 shows a plot of 1/(*C_m_—C_s_*) versus thickness as measured for specimens of V–4 Brazil Ruby mica and for V–2 India Ruby mica. A fit of the data gives 425 pf for *C_a_.* Both *C_s_* and *C_m_* showed less than 1 percent change with frequency over the range considered.

The loss tangent, *D_x_=ωR_x_C_x_*, for the mica crystal can be derived straightforwardly from the equivalent circuit. With the assumption that *D_x_*≪1 and with eq (1),

$$D_{x}=\frac{D_{m}C_{m}C_{a}}{\left\{C_{a}-C_{m}+C_{s}\right\}\left\{C_{m}-C_{s}\right\}}.$$ (2)

**Figure 4:**
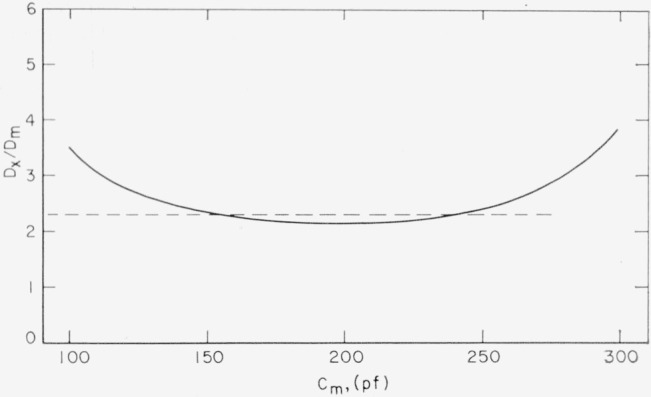
Correction factor *D_x_*/*D_m_* as a function of *C_m_*. *D_m_* is measured loss tangent; *D_x_* is that for mica proper. *C_m_* is total measured capacitance.

The functional dependence of the ratio *D_x_*/*D_m_* on *C_m_* is shown in figure 4. Experimental effort was limited to the central region of the dependency where a reasonable approximate value of the ratio has been taken as 2.3.

### 2.4. Behavior of Loss Tangent

Typical loss tangent data are given in table 1 for measurements made on a sample of V–2 India Ruby mica at 1000 c/s and at ~40 percent. The sample was first measured as received. It was then removed, and a thin lamina was cleaved from one side. Each resultant piece, thick and thin, was then examined with the apparatus. The freshly peeled surface was placed first against the small plate and then against the large plate. As can be seen peeling has caused a very large increase in the measured value of loss tangent and a strong orientation effect.

**Figure 5:**
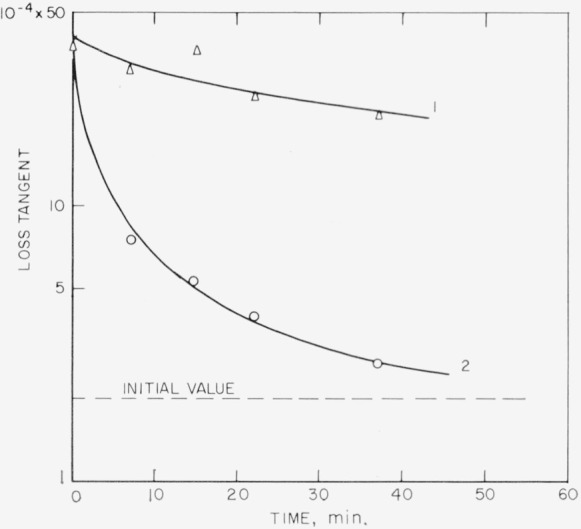
Decay in loss tangent after cleavage at room temperature. The original specimen was cleaved into two laminae of equal thicknesses. One was then held at room temperature (curve 1). The second was ovened at 125 °C (curve 2). Ambient Rh of 50%. Frequency of 1000 c/s.

Measurements were made to determine the change in loss tangent with time. Results of aging at two temperatures for room ambient rh~50 percent and for 1000 c/s are shown in figure 5. For these data the original sample was cleaved into two approximately equal thicknesses. For the higher temperature, the sample was then aged in an oven and removed only long enough for each measurement. Two to three minutes were needed for each of these measurements, during which time the sample had cooled to room temperature (as determined with a similar specimen and a fine thermocouple placed between laminae).

**Figure 6:**
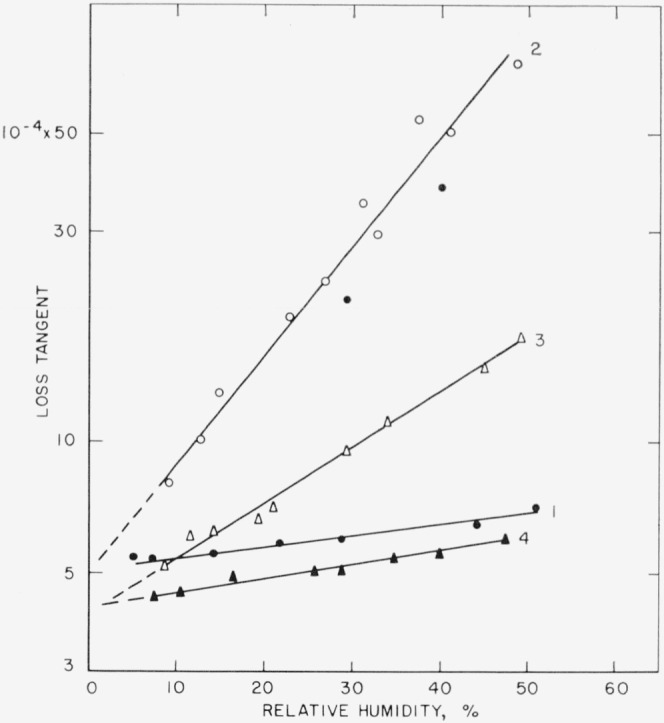
Measured loss tangent as a function of *Rh*. 1. Initial values before splitting, (thickness of 12 × 10^−3^ in.). 2. Cleaved face to small plate (thickness of 10 × 10^−3^ in.). Solid points represent values obtained with decreasing humidity. 3. Cleaved face to large plate. 4. Layer of petrolatum over cleaved face and cleaved face to small plate (thickness of 9.4 × 10^−3^ in.). Frequency of 1000 c/s.

The dependence of the loss tangent on the relative humidity is displayed in figure 6. The procedure used to obtain these data was as follows. A thin lamina was cleaved from the original specimen at an rh of <10 percent at room temperature. The remaining thick section was oriented with the freshly peeled face to the small electrode. Relative humidity was increased in stages to about 50 percent and *D* was measured after each increment. *D* responded quickly to change in rh. Steady state occurred within 2 min after each change. The rh was quickly returned to <10 percent, the section was turned over to orient the freshly peeled face to the larger plate, and the cycle was repeated. Then a layer of petrolatum was spread over the fresh surface, the sample was reinserted with freshly peeled face to small electrode, and the data of curve 4 resulted. Each cycle was of ½ hr duration. The data indicate that *D* increases exponentially with rh as

$$\log\;D-\log\;D_{0}=a\cdot \text{rh}.$$ (3)

The value of the slope, *a*, depends upon the orientation of the freshly peeled surface.

## 3. Theory

Let us assume the capacitor is composed of two regions, the first region being that under the small electrode where the *E* field is always normal to the dielectric plane, and the second region being the edge where fringing occurs in the *E* field. The loss tangent for the holder and specimen is by definition the ratio of the resistive and reactive components of the total current, *I*, passing between the electrodes. The total current is the sum of that under the small plate, *I*_plate_, and that at the edge, *I*_edge_, and the corresponding loss tangent, *D_t_*, is

Dt=Re[Iplate]Im[Iplate+Iedge]+Re[Iedge]Im[Iplate+Iedge].

Assuming *I*_edge_ ≪ *I*_plate_, we have

$$D_{t}=D_{s}+D_{e},$$ (4)

where

Ds=Re[Iplate]Im[Iplate]

and

De=Re[Iedge]Im[Iplate]. (5)

### 3.1. Region Within the Normal Field

Although the surface film may itself be multilayered or otherwise complicated, it is here considered as a single layer for simplification. The material is thus treated as a two layered dielectric, mica and surface film, as shown in figure 8, where each is considered as a parallel combination of a resistance and capacitance. The loss tangents for the film and the mica are respectively

$$\begin{array}{l} D_{1}=\frac{1}{R_{1}\omega C_{1}},\\ D_{2}=\frac{1}{R_{2}\omega C_{2}}. \end{array}$$ (6)

The loss tangent for the combination, obtained from the ratio of the real to the imaginary components of the total impedance, is

$$D_{s}=\frac{R_{1}\left[{\left(R_{2}\omega C_{2}\right)}^{2}+1\right]+R_{2}\left[{\left(R_{1}\omega C_{1}\right)}^{2}+1\right]}{R_{1}^{2}\omega C_{1}\left[{\left(R_{2}\omega C_{2}\right)}^{2}+1\right]+R_{2}^{2}\omega C_{2}\left[{\left(R_{1}\omega C_{1}\right)}^{2}+1\right]}.$$ (7)

Assuming *R*_2_*ωC*_2_≫1 (i.e., *D*_2_≪1) and *C*_1_≫*C*_2_, eq (7) becomes

$$D_{s}≅\frac{R_{1}\omega C_{2}}{1+{\left(R_{1}\omega C_{1}\right)}^{2}}+D_{2}.$$ (8)

This last expression has the frequency dependence of a relaxation process. It further indicates that the increase in loss tangent due to the surface film is a direct function of the capacitance of the bulk mica and, hence, is a function of the thickness of the mica specimen. If a specimen is cleaved into two samples of thicknesses *t*′ and *t*″ having identical surface films, then by eq (8)

$$D_{s}^{\prime }-D_{2}=D^{\prime }\propto \frac{\epsilon_{2}}{t^{\prime }},$$ (9)

and

$$D_{s}^{\prime \prime }-D_{2}=D^{\prime \prime }=\frac{t^{\prime }}{t^{\prime \prime }}D^{\prime }.$$ (10)

Thus, if after cleavage the two resultant samples are measured with the cleaved faces toward the larger electrode, the loss tangent of the thin section should be greater than the thick by the ratio of the thicknesses.

### 3.2. Region of the Fringe Field

Consider the region at the edge to be a distributed constant radial transmission line. Let it be composed of a number of parallel sections of unit width— a good approximation because of the small extent of the fringe pattern. For the present case, with the assumption of a sufficiently large surface conductivity, most of the fringe field will be confined to the resistive film and the dielectric slab. The potential applied to the terminals of the transmission lines is just that between the capacitor plates. The situation is pictured in figure 9. The fringe field is of short extent and the attenuation is therefore great enough so that only the outgoing wave need be considered, i.e., no termination and consequent reflection. The resultant current determines the edge loss tangent, *D_e_.* The current, *J*, down the unit width line is *V*/*Z*_0_, where $Z_{0}=\sqrt{Z_{s}Z_{p}}$ is the characteristic impedance, *Z_s_* is the series impedance/unit length, and *Z_p_* is the parallel or shunt value/unit length [15]. With *R*′ as the surface resistance/unit square and *C*′ as the capacitance/unit square, which is taken identical to that between the plates (i.e., *C*_2_/*A* with *A* being the area of the small electrode)

$$Z_{0}\equiv \sqrt{Z_{s}Z_{p}}=\sqrt{-\frac{iR^{\prime }}{\omega C^{\prime }}},$$ (11)

and

$$J=V/Z_{0}=V\frac{1+i}{\sqrt{2}}\sqrt{\frac{\omega C^{\prime }}{R^{\prime }}}.$$ (12)

By eq (5) *D_e_* is the ratio of the real component of the edge current to the total capacitative current. Therefore,

De=2πrRe(J)Ic=2r12R′ωC′∝2rt2R′ωϵ′, (13)

where *I_c_≅ωC*′*AV* and *r* is the radius of the small electrode. For two thicknesses *t*′ and *t*″ cleaved from the specimen,

$$D_{e}^{\prime }=\sqrt{\frac{t^{\prime }}{t^{\prime \prime }}}D_{e}^{\prime \prime },$$ (14)

where $D_{e}^{\prime }$ and $D_{e}^{\prime \prime }$ are the edge dissipation factors for the two thicknesses.

## 4. Experimental Verification of Expressions for Loss Tangent

### 4.1. Loss Tangent and Thickness

The experimentally determined values are (1) the initial uncleaved value or ~*D*_2_, (2) the value for the cleaved face against the large electrode or *D_s_*, and (3) the value for the cleaved face against the small electrode or *D_t_*, the total loss tangent. Each of these is obtained from the actually measured values after correction for airgap effect. Thus, the expected value of loss tangent for a thin section, $D_{t}^{\prime \prime }$, as compared to its complementary thick section $D_{t}^{\prime }$, is by eqs (4), (9), (10), and (14)

$$D_{t}^{\prime \prime }=n\left(D_{s}^{\prime }-D_{2}\right)+D_{2}+\frac{D_{t}^{\prime }-D_{s}^{\prime }}{\sqrt{n}},$$ (15)

where *n* is the ratio *t*′*/t*″.

A number of crystals were split in such a way that a variety of values of *n* were achieved. Table 2 lists results comparing the experimental value of loss tangent for the thin section to the value predicted from the data of the complementary thick section. Since the correction factor for airgap effect should be applied to each of the terms of eq (15) and hence is common, data are given as the actually measured values.

The ratios of measured to predicted values show an average difference from unity of about 17 percent and 15 percent for *D_s_* and *D_t_* respectively. These are reasonable values. In addition to the assumptions made for analytical purposes, it was also assumed that the surface states were the same in each complement after cleavage. This may not be strictly true. On the other hand, it is to be remembered that the surface is very sensitive to handling and contact [5]. Each complement had to be handled separately so that the chance always existed of introducing a discrepancy. Further, elapsed time between measurements for each section allowed *D* to undergo a relative decay (see fig. 5).

### 4.2. Extent of Fringe Field

The current falls to 1/*e* of its initial value at a distance down the transmission line equal to the reciprocal of the attenuation constant, *α.* This then is a measure of the extent of the fringing field. Since [15]

α=Re[Zs/Zp]=ωR′C′2,

by eq (13)

$$\alpha =\frac{1}{rD_{e}}.$$ (16)

From the data of figure 6 and with eq (4), the corrected values of *D_e_* range from about 16×10^−3^ at 50 percent rh down to about zero at 0 percent rh. Thus, for the dielectric slab of 0.254 mm thickness the computed attenuation distance ranged from about 0.2 mm down to about zero, with a value of 0.01 mm at 10 percent rh. The significance of zero attenuation distance for this model (see fig. 9) is that the surface resistance per square (*R*′) is infinite and the fringe field is reduced to the normal capacitive situation.

Upon the assumption that the field is essentially contained between the surface conducting film and the opposite electrode, a guard ring structure would show little effect until the guard-electrode gap became comparable to the attenuation distance. As a check on this, a capacitor was made with tin foil electrodes. A 1 in. diam electrode and a guard ring with a 0.010 in. guard gap were formed, rolled out on a sheet of plate glass, greased with petrolatum, heated at 100 °C, lifted from the glass, and rolled carefully onto the cleaved face of a 0.010 in. thick mica crystal, first without the guard and then with the guard. Care was taken that only a thin film of petrolatum remained on the foil and did not spread into the guard-electrode gap. The rh was varied and loss tangent measurements were made. The measured loss tangent ranged from 8×10^−4^ at 8 percent rh to 30×10^−4^ at 50 percent rh and was not diminished when the guard ring was added. Then, a second capacitor plate system was constructed. This comprised a thick center electrode of 1.650 in. diam and ⅜ in. thickness, a guard ring of the same thickness but of 0.300 in. width, and a guard-to-plate gap of 0.0025 in. The cleaved specimen was 16.3 ×10^−3^ in. in thickness. The effect of rh on *D* is displayed in figure 10. The difference in value with and without the guard is considerable, but the slopes of the loss tangent-rh dependencies are the same down to a low rh of ~15 percent. The extent of the fringe field was apparently affected, as *D* was diminished by the guard, but the tangential surface interaction persisted. For if the tangential surface interaction were voided, one would expect a slope indicative of normal field only.

## 5. Nature of the Surface Film

**Figure 7:**
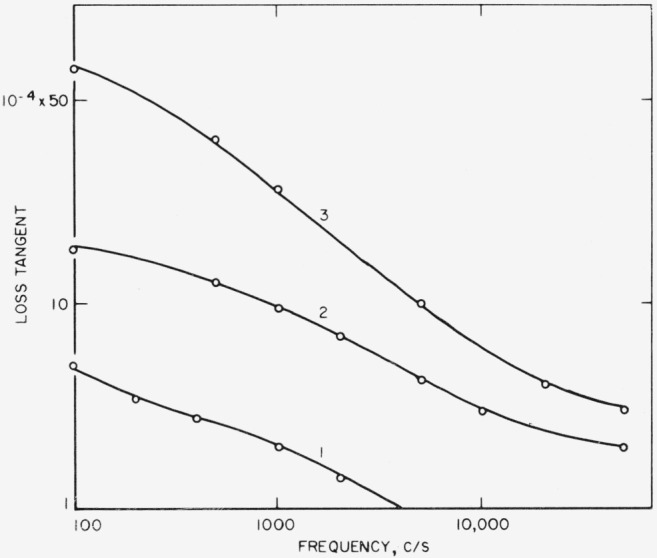
Measured frequency dependence of loss tangent for both orientations of peeled surface. 1. Before splitting. 2. After splitting, cleaved side to large plate. 3. Cleaved side to small plate. Rh, 40%. Thickness after peeling, 0.010 in.

*D*_1_ *R*_1_ and *C*_1_ (sec. 3.1) can be computed from eq (8) and frequency characteristics such as those of figure 7. Equation (8) has the form

$$D_{s}-D_{2}≅\frac{\xi f}{1+{\left(\beta f\right)}^{2}},$$ (8′)

where ξ = 2*πR*_1_*C*_2_, *β* = 2*πR*_1_*C*_1_, and *f* is the frequency. We make the approximation that within a small frequency interval *β* and ξ remain constant; e.g., 100 to 200 c/s at one end of the spectrum and 5000 to 10,000 c/s at the other. This allows solution for *β* and ξ with the values for *D_s_* and *D*_2_ at the two endpoints of the frequency interval, i.e., two simultaneous equations. *D*_1_ is then $1/\beta \bar{f}$, where $\bar{f}$ is the mean frequency, and *C*_1_ is related to *C*_2_ by the ratio *β*/ξ. For this *D*_2_, *D*_s_, and *C*_2_ are first corrected for airgap effect. Results obtained for *D*_1_ for successive frequency intervals from the data of figure 7 are given in figure 11 at the mean frequency along with the loss tangents of conductivity water, ice, and fresh snow [16]. Results for *R*_1_ and *C*_1_ are given in figure 12. Quantities are all reduced to values per cm^2^, e.g., pf/cm^2^ and Ω/cm^2^ in the direction normal to the surface.

*D_e_* is obtained from figure 7 by eq (4). *R*′ is then found by eq (13). Results for *D_e_* and *R*′ are given in figure 13.

These results indicate that the frequency characteristic of *D*_1_ is not that of pure bulk water or ice. It appears to be more like that of fresh snow, a dispersed structure. The characteristics of *R*_1_ and *D_e_* also seem reasonable.

**Figure 8:**
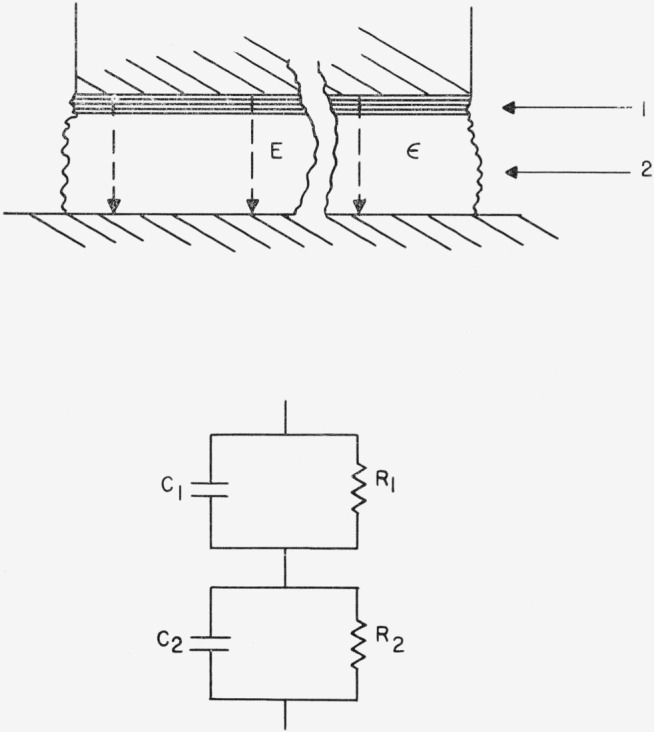
Representation of the area under the plates of the capacitor where the field is normal to the dielectric plane. *C*_1_ and *R*_1_ represent the adsorbed layer, while *C*_2_ and *R*_2_ represent the mica crystal.

**Figure 9:**
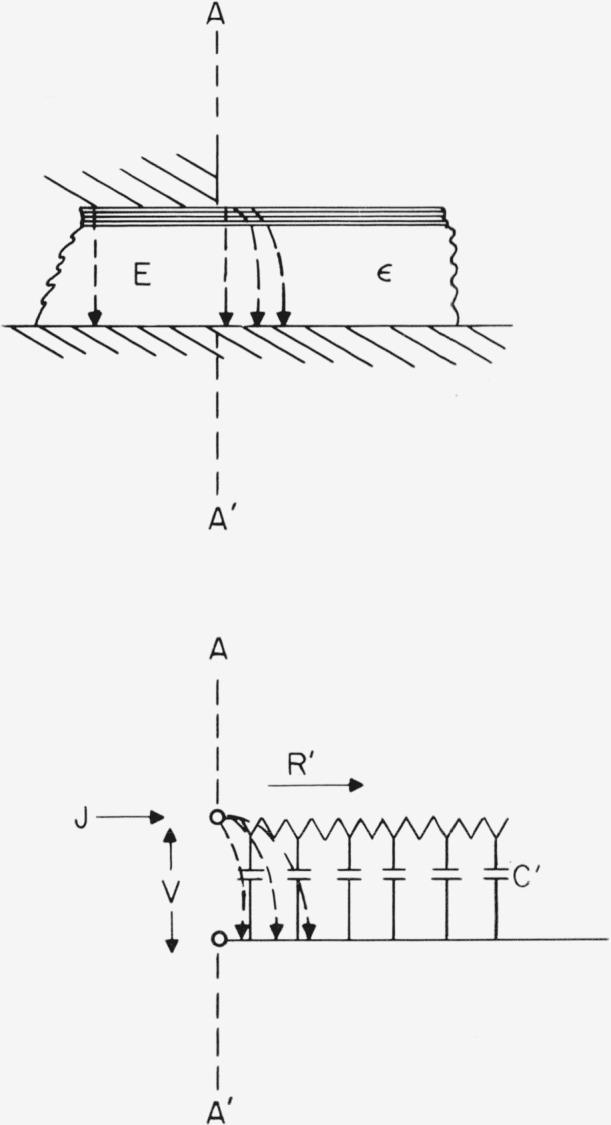
Representation of the fringe field at the edge of the capacitor by a transmission line. The parallel resistance per square of the surface film is *R*′. The capacitance per cm^2^ of the mica is *C*′.

**Figure 10:**
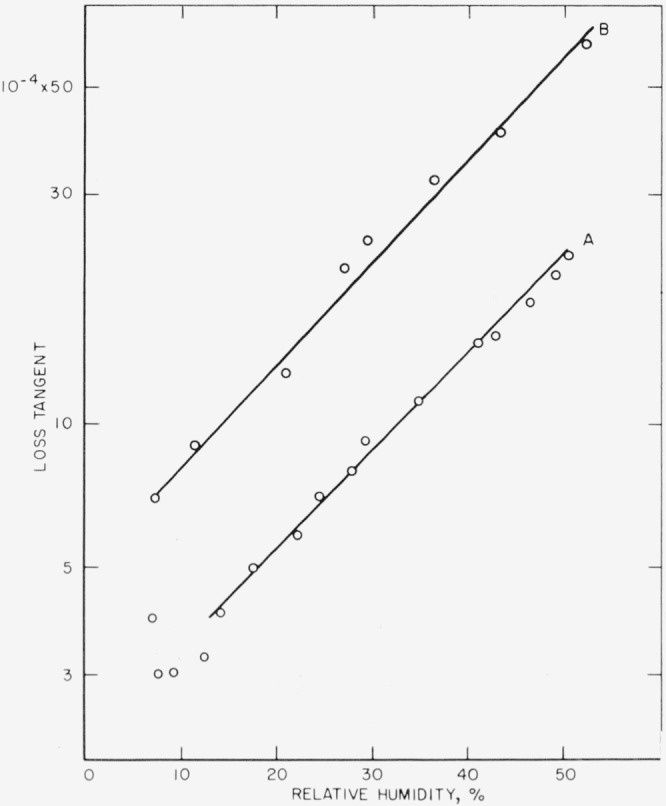
Effect on loss tangent of a closely spaced guard ring for a cleaved sample as a function of *Rh*. Thickness is 16.3×10^−3^ in. Guard-to-plate gap is 2.5×10^−3^ in. Center plate diameter is 1.6 in. A. with guard. 13. without guard. Frequency of 1000 c/s.

**Figure 11:**
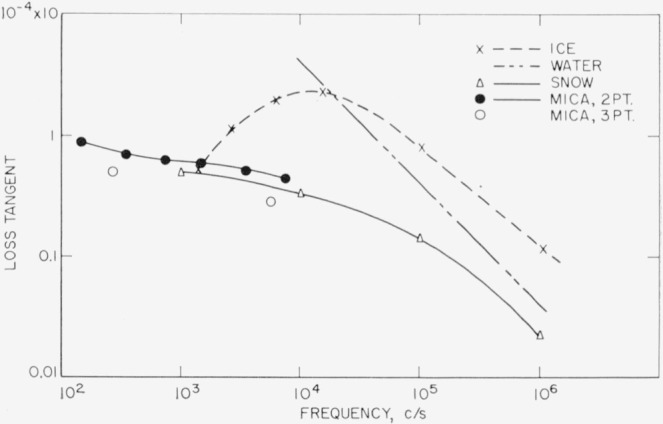
Loss tangent versus frequency for the surface film on mica as derived by 2 and 3 point analyses (from the data of figure 7) and for the several forms of water: conductivity liquid at 25 °C, ice, and snow (see ref. 16).

**Figure 12:**
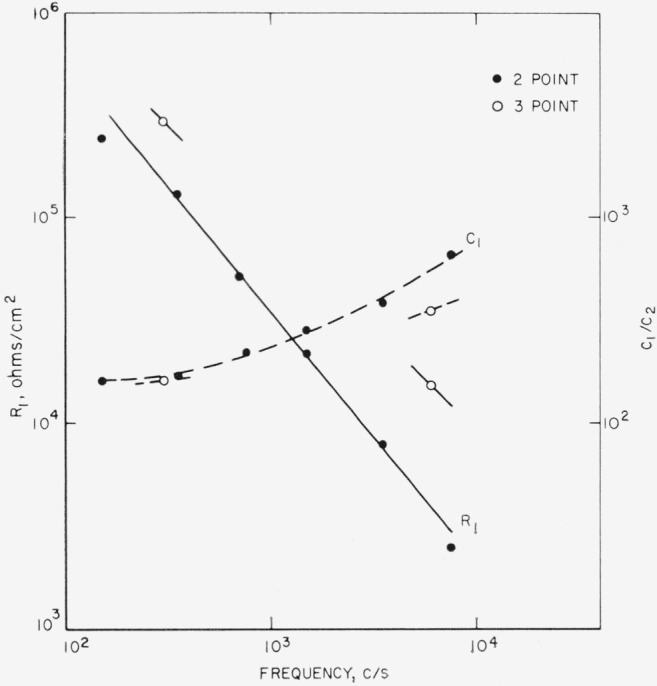
*R*_1_ and *C*_1_/*C*_2_ versus frequency as derived from 2 and 3 point analyses for a Tanganyika Ruby mica from the data of figure 7.

**Figure 13:**
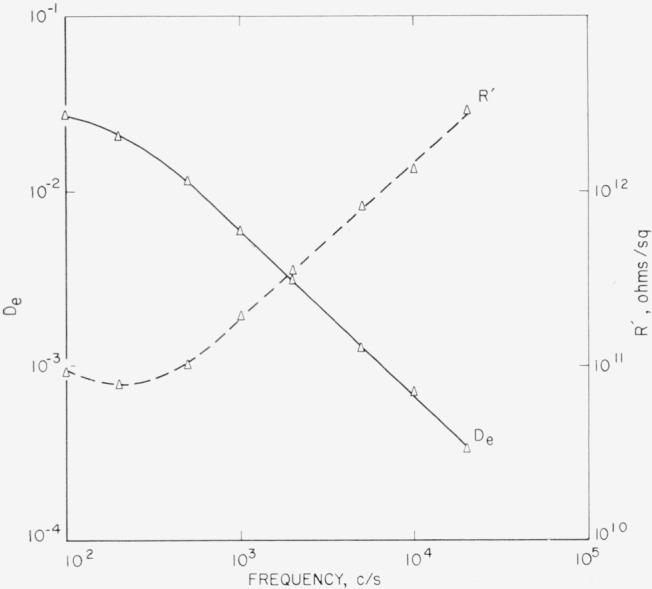
Loss tangent at the edge and resistance along the surface as a function of frequency for a Tanganyika Ruby mica as derived from the data of figure 7.

**Figure 14:**
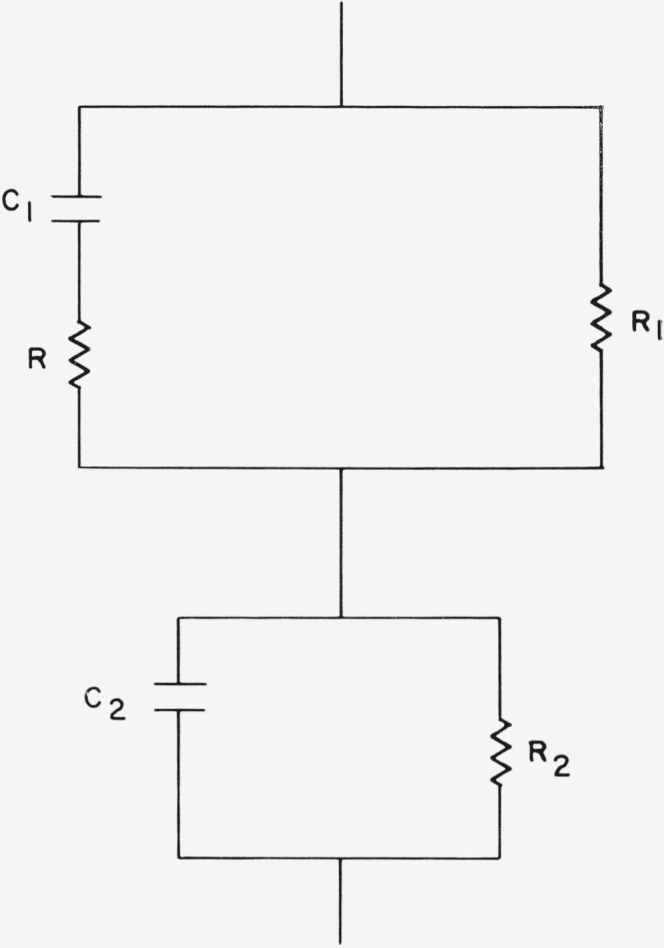
Modified equivalent circuit with the adsorbed film represented by an empirically suggested circuit for pure water (see Von Hippel, ref. 16).

The derived natures *C*_1_ and *R*′ raise the question as to whether such behavior is intrinsic or only due to oversimplification of equivalent circuits. Therefore, the equivalent circuit for the surface film in the normal direction (fig. 8) was modified by the addition of a series resistance to *C*_1_ (see fig. 14). For example, such an equivalent circuit has been suggested as an empirical fit for pure water [17]. The resultant equations for the complete circuit are given in appendix A. Some numerical results have been included in figures 11 and 12, where they are labeled as 3-point data (computation now requires three data points within any chosen frequency interval). As indicated the behaviors of the quantities are essentially as before, although *C*_1_ now shows a smaller frequency dependence. It is assumed from this that further circuit modification would lead to constant *C*_1_ in this frequency range but that the magnitudes of the quantities are essentially as given. Presumably then, *D*_1_ has a characteristic different from that of a sheet of liquid water or of ice and more like that of a dispersed structure.

The assumption of a uniform surface film is inconsistent with the magnitudes of the derived quantities. For example, from figure 12 at 1000 c/s *C*_1_ has the value of 6×10^−9^ f/cm^2^ (*C*_2_=30 pf/cm^2^), which, if *ϵ*_1_ were 1, would lead to a film thickness of about 1300 A. If *ϵ*_1_ were greater than unity, the thickness would have to be correspondingly greater. But, a film of this thickness is unreasonable [5, 18]. Similar inconsistencies arise in the values of *R*_1_ and *R*′. As *R*′ is the resistance along the plane of a cm^2^ of thickness, *t*, and *R*_1_ is the resistance normal to the plane for the same section, i.e., *R*′ *= ρ_p_*/*t* while *R*_1_*=ρ_n_t*, where *ρ_n_* and *ρ_p_* are the resistivities normal to and parallel to the plane respectively,

$$R_{1}R^{\prime }=\rho_{n}\rho_{p}.$$

If *ρ_n_* and *ρ_p_* are taken as equal, then *ρ* is found to be ~10^8^ Ω cm at 1000 c/s where *R*′ = 2×10^11^ Ω and *R*_1_ = 3 × 10^4^ Ω. But, from this Value for *ρ, t* would have to have been >30,000 Å as computed from either of the expressions for *R*_1_ and *R*′. To bring *t* to within a reasonable limit as computed from *R*_1_ and from *R*′, *ρ_n_* should be orders of magnitude greater than 10^8^ Ω cm and *ρ_p_* should be correspondingly less than 10^8^ 12 cm. Thus, the resistivity in the normal direction must be considerably greater than that along the surface. In fact, a factor of *ρ_n_* to *ρ_p_* of more than 100 would be required to bring *t* to within reasonable limits [18]. This, however, conflicts with the derived values of *D*_1_. Since

$$D_{1}≅\frac{1}{R_{1}\omega C_{1}}=\frac{2}{\rho_{n}\epsilon f}\cdot 9\times 10^{11},$$ (17)

a value for *D*_1_ of 0.5 at 1000 c/s with *ϵ* of 1 leads to a *ρ_n_* of ~4×10^9^ Ω cm. This is only a factor of 40 over 10^8^. Any larger increase in *ρ_n_* as, say, a factor >100, would cause *D*_1_ to be less than the derived values.

A conclusion from these results is that the surface film has a more complex structure than has been suggested [5]. One possibility is that the adsorbed layer is composed of small islands or patches. Such a formation would also be compatible with the observation of alternately electrically charged areas on the surface of cleaved mica [3, 4]. Then with the smaller area, *C*_1_ would be smaller than for a continuous film, *R*_1_ would be larger, and yet *D*_1_ would remain the same as this is only dependent on the material according to eq (17). The compatibility of the patch formation and the limitations imposed are discussed in appendix B as based upon suitable equivalent circuit analysis.

Lastly, from eq (8) and the derived magnitude of *D*_1_ (see fig. 11), the change in *D_s_—D*_2_ is essentially that due to the change in *R*_1_. Since *D_s_—D*_2_ increased with rh, according to the patch concept the islands of loosely bound film must increase in depth with rh rather than expand in diameter in order to cause *R*_1_ and hence *D_s_—D*_2_ to increase. Such formation would be sensitive to rh as is the case.

## Figures and Tables

**Figure 15:**
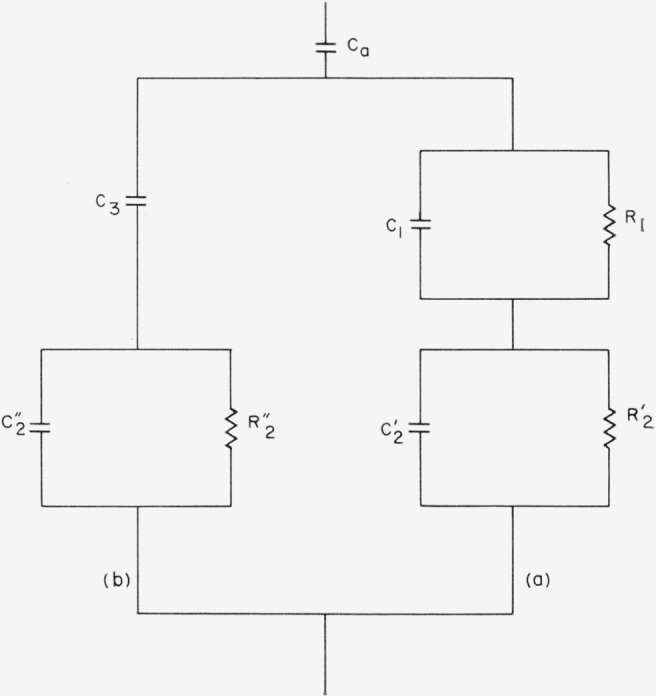
Equivalent circuit for patch formation on the surface of peeled mica.

**Table 1:** Measured loss tangent for cleaved sections with fresh face oriented first to large and then to small electrode for a V–2 India Ruby at 1000 c/s Rh of ~40%

	Loss tangent
1. Original sample, 0.267 mm thick	<0.0001
2. Losses of thick layer 0.224 mm thick	
a. Fresh surface against large electrode	.0010
b. Fresh surface against small electrode	.0072
3. Losses of thin layer 0.043 mm thick	
a. Fresh surface against large electrode	.0060
b. Fresh surface against small electrode	.0080

**Table 2:** Testing of derived expressions of loss tangents by comparing predicted to experimental values for thick and thin complements

1. *n*	2. *D*′*_s_–D*_2_	3. *D*″*_s_–D*_2_	4. *D*′*_t_–D*_2_	5. *D*″*_t_–D*_2_	$6.\frac{D^{\prime \prime }{\;}_{s}}{D^{\prime \prime }{\;}_{s}\left(\text{predicted}\right)}$	$7.\frac{D^{\prime \prime }{\;}_{t}}{D^{\prime \prime }{\;}_{t}\left(\text{predicted}\right)}$
						
1.20	8.1×10^−4^	10.4×10^−4^	69×10^−4^	64×10^−4^	1.06	1.00
1.8	3.6	8.4	76	67	1.29	1.06
3.3	9.1	35	57	75	1.18	1.33
4.5	9.9	59	74	87	1.32	1.17
4.7	7.3	42	54	69	1.21	1.23
8.2	7.1	50	64	100	0.85	1.28
9.0	16.0	150	86	164	1.04	0.98
15.6	9.2	121	82	145	0.84	.90

Notes:

Rh~40 percent. Frequency is 1000 c/s.

Thickness measured by direct reading micrometer for specimens of thickness >0.002 in. and by dial comparator (least reading of 0.00002 in.) for specimens of thickness ≤0.002 in. Cleaved sections often have faults causing thickness changes of <0.0001 in. magnitude as well as wedge shaped cross sections. Larger *n* values are subject to consequent errors of several percent.

**Table 3:** Derived values for loss tangent and equivalen circuit dements through 3-point analysis*

*f*^a^	$\bar{f}$^b^	*ξ*	*ß*	*R* Ω	*C*_1_/*C*_2_	*R*_1_ Ω	*D*_1_
							
*c/s*	*c/s*						
1. 10.000							
2. 5,000	5700	2×10^−6^	6.9×10^−4^	67	350	10^4^	0. 28
3. 2,000							
1. 500							
2. 200	270	5.5×10^−5^	8.8×10^−3^	9900	160	2.9×10^5^	0.5
3. 100							

* Values listed are per cm^2^.

a *f* represents the frequency of the 3 data points chosen in the frequency interval considered.

b $\bar{f}$ represents the approximate average frequency of the 3 points, *f*.

## Footnotes

1 Figures in brackets indicate the literature references at the end of this paper.

## 6. Appendix A. Modified Equivalent Circuit and 3-Point Analysis

The equivalent circuit for the surface film is now taken as one which has been suggested for liquid water [17]. The resultant equivalent circuit for film and mica is shown in figure 14. The admittance for the film itself is

$$Y_{1}=\frac{1}{R_{1}}+\frac{R{\left(\omega C_{1}\right)}^{2}}{1+{\left(R\omega C_{1}\right)}^{2}}+i\frac{\omega C_{1}}{1+{\left(R\omega C_{1}\right)}^{2}}.$$ (18)

In terms of the complex dielectric constant, *ϵ** = *ϵ*′—*iϵ*″, and with *Y*_1_ ***≡*** *iωC*,

$$Y_{1}=\omega \epsilon^{\prime \prime }\frac{C_{0}}{\epsilon_{0}}+i\epsilon^{\prime }\frac{\omega C_{0}}{\epsilon_{0}},$$ (19)

where *C*=(*ϵ*/ϵ*_0_)*C*_0_ and *C*_0_ is the equivalent free space capacitance of the film. Thus

$$\epsilon_{1}^{\prime }=\epsilon_{0}\cdot \frac{C_{1}}{C_{0}}\cdot \frac{1}{1+{\left(R\omega C_{1}\right)}^{2}}.$$ (20)

The loss tangent for the film is

$$D_{1}=\frac{1+R{\left(\omega C_{1}\right)}^{2}\left(R+R_{1}\right)}{R_{1}\omega C_{1}}.$$ (21)

On the expectation that *R* is sufficiently smaller than *R*_1_ i.e., *R<R*_1_,

$$D_{1}≅\frac{1}{R_{1}\omega C_{1}}+R\omega C_{1}.$$ (22)

With the same assumption that *R < R*_1_, the loss tangent for the double layer is

$$D_{s}=\frac{\left[R_{1}^{2}R\omega^{2}C_{1}^{2}+R_{1}\right]\left[{\left(R_{2}\omega C_{2}\right)}^{2}+1\right]+R_{2}\left[{\left(R_{1}\omega C_{1}\right)}^{2}+1\right]}{R_{1}^{2}\omega C_{1}\left[{\left(R_{2}\omega C_{2}\right)}^{2}+1\right]+R_{2}^{2}\omega C_{2}\left[{\left(R_{1}\omega C_{1}\right)}^{2}+1\right]}.$$ (23)

Then, under the assumption that *R*_2_*ωC*_2_≫1 and *C*_1_ ≫ *C*_2_,

$$D_{s}≅\frac{R_{1}\omega C_{2}}{1+{\left(R_{1}\omega C_{1}\right)}^{2}}+D_{2}+\frac{R{\left(R_{1}\omega C_{1}\right)}^{2}\omega C_{2}}{1+{\left(R_{1}\omega C_{1}\right)}^{2}}.$$ (24)

This has the form

$$\frac{D_{s}-D_{2}}{2}\left[1+{\left(\beta f\right)}^{2}\right]=\xi +2\pi R{\left(\beta f\right)}^{2}C_{2},$$ (25)

where again ξ = 2*πR*_1_*C*_2_ and *β* = 2*πR*_1_*C*_1_. Similarly as before *β, ξ*, and *R* are considered constant over a given frequency interval. *D_s_, D*_2_, and *f* are now obtained from three data points in the given frequency interval which allow three simultaneous equations to be solved for *β*, ξ, and *R.* Data for two such frequency intervals as taken from figure 7 are listed in table 3 and included in figures 11 and 12.

It is seen that *D*_1_ is slightly smaller than for the 2-point treatment, and *R* is 1 to 3 percent of *R*_1_. The term *RωC*_1_ has the value of ~0.03 to ~0.08. Therefore, the second term of eq (22) is some 15 to 20 percent of the first term. In eq (20) (*RωC*_1_)^2^ may be neglected so that *C*_1_ is again a measure of $\epsilon_{1}^{\prime }$. The second term on the right of eq (25) is 10 to 15 percent of ξ.

## 7. Appendix B. Patch Formation

The previous analyses considered the normal *E* field region of the test capacitor as an airgap in series with the adsorbed film and mica substrate, i.e., the equivalent circuit of figure 8 in series with the airgap capacitance, *C_a_* However, in the case of small and numerous patches adsorbed on the mica surface we must consider two regions of the *E* field. In one region *E* lines pass through an airgap directly into the mica substrate. In the second, *E* lines pass through an airgap into the patches, in which case there may be some refraction. Such a situation becomes that of figure 15, where *C_a_* now represents an airgap of thickness equal to the distance from the capacitor plate to the top of a patch, *R*_1_ and *C*_1_ represent the patches, $R_{2}^{\prime }$ and $C_{2}^{\prime }$ represent the mica for the area under the patches, *C*_3_ is the air film of thickness equal to the height of the patches, $R_{2}^{\prime \prime }$ and $C_{2}^{\prime \prime }$ represent the mica under the air film. Similarly to before, eq (4), for the region below the airgap, *C_a_*,

D=Re[Ia+Ib]Im[I]=Re[Ya]Im[Y]+Re[Yb]Im[Y], (26)

where Im [*Y*] is essentially *ωC*_2_. Then for branch (a) of figure 15 with $C_{1}\gg C_{2}^{\prime }$, $\omega R_{2}^{\prime }C_{2}^{\prime }\gg 1$, and $R_{2}^{\prime }>R_{1}$ the expression for *D_a_* becomes identical in form to that of eq (8), or

$$D_{a}≅\frac{1}{\omega R_{2}^{\prime }C_{2}}+\frac{R_{1}\omega C_{2}^{\prime 2}}{1+{\left(\omega R_{1}C_{1}\right)}^{2}}\cdot \frac{1}{C_{2}}.$$ (27)

For the second term of eq (26) we have, with $C_{3}\gg C_{2}^{\prime \prime }$,

$$D_{b}≅\frac{1}{\omega C_{2}}\cdot \frac{\omega^{2}R_{2}^{\prime \prime }C_{3}^{2}}{1+{\left(\omega R_{2}^{\prime \prime }C_{3}\right)}^{2}}.$$

With multiplication of $\omega R_{2}^{\prime \prime }C_{3}$ by $C_{2}^{\prime \prime }/C_{2}^{\prime \prime }$ and as $\omega R_{2}^{\prime \prime }C_{2}^{\prime \prime }=\frac{1}{D_{2}}\gg 1$,

$$D_{b}≅\frac{1}{\omega R_{2}^{\prime \prime }C_{2}}.$$ (28)

The limiting behavior of the patches can now be seen from eq (27) and (28). The first limit is that for which the *E* field does not deviate, i.e., there is no refraction at the patches and all field lines are parallel to each other. Then $C_{2}^{\prime }$ is the capacitance of the mica area directly under the patches, or if *A* is the patch area/cm^2^,

$$C_{2}^{\prime }=C_{2}\cdot A$$

and

$$C_{2}^{\prime \prime }=C_{2}\left(1-A\right).$$

Since

$$\frac{1}{\omega R_{2}^{\prime }C_{2}^{\prime }}=D_{2}=\frac{1}{\omega R_{2}^{\prime \prime }C_{2}^{\prime \prime }},$$

the loss tangent is

$$D=D_{2}+A\frac{R_{1}\omega C_{2}^{\prime }}{1+{\left(R_{1}\omega C_{1}\right)}^{2}}.$$ (29)

In this case as *A* becomes small, *D→D*_2_. But this also means that when *D* is equated to the derived values of *D_s_*, $R_{1}\omega C_{2}^{\prime }/\left[1+{\left(R_{1}\omega C_{1}\right)}^{2}\right]$ must be correspondingly greater by 1/*A* than the value determined by eq (8) so that *R*_1_ would be found to be even greater than before and $C_{1}^{\prime }$ even less. This compounds the difficulties already established.

The other limiting case is that for which refraction does occur in the *E* field to the extent that all *E* field passes through the patches before entering the mica. For this case $C_{2}^{\prime }=C_{2}$, $C_{2}^{\prime \prime }=0$, $R_{2}^{\prime \prime }\to \infty$ and we again have eq (8) from eqs (27) and (28). To realize this limit physically, the patches would have to be small in area, be very numerous, be of sufficient height to cause distortion of *E*, and have sufficient dielectric constant.

It is thus possible for the patch concept to give the derived values. The values of *R*_1_ can be larger and those of *C*_1_ can be smaller than for a continuous film; yet, the adsorbed layer character of resistivity, dielectric constant and thickness need not be abnormal. We cannot now equate *R*_1_ to *ρ_n_t*/*A*, however, for there are for this case tangential components of the field within the patch, although obviously not of the order of those of the fringing field at the plate edge. One would expect a situation somewhere between these two extremes but closer to the second, since *D_s_~*1/*t* rather than $\sqrt{t}$.
